# A Qualitative Study to Explore Global Trends in Clinical Pharmacy Practice

**DOI:** 10.1111/jep.70104

**Published:** 2025-05-05

**Authors:** Laura Alexandra Moura Gomes, Adriano Miguel Gomes Costa, Stephane Marc Richard Steurbaut, Helder Dias Mota Filipe, Silvana Nair Leite, Filipa da Palma Carlos Alves da Costa Azevedo e Silva

**Affiliations:** ^1^ Research Institute for Medicines (iMED.ULisboa), Faculty of Pharmacy University of Lisbon. Av. Prof. Gama Pinto Lisboa Portugal; ^2^ Farmácia Cunha Barcelos Portugal; ^3^ Department of Hospital Pharmacy Vrije Universiteit Brussel (VUB), Universitair Ziekenhuis Brussel (UZ Brussel) Brussels Belgium; ^4^ Federal University of Santa Catarina: Florianopolis, R. Eng. Agronômico Andrei Cristian Ferreira, s/n ‐ Trindade Florianópolis SC Brasil

**Keywords:** career choice, clinical pharmacy, hospital, leadership, motivation, patient care team, pharmacists, pharmacy service

## Abstract

**Rationale:**

Pharmacists' clinical responsibilities have been on a progressive rise, adopting a more person‐centered approach, and experiencing increased involvement in healthcare teams across all settings. The development and evolution of clinical pharmacy practice in each country depends on the existing facilitators and barriers, which are variable and highly context‐related.

**Aims and Objectives:**

To investigate the perspectives of global experts regarding the advancement of clinical pharmacy practice, with a specific focus on examining the factors contributing to success and their outlook on the future of clinical pharmacy.

**Methods:**

Semi‐structured interviews were conducted with academia and practice experts from 13 countries. Each expert was asked what they considered to be the future of clinical pharmacy education and practice, as well as what should be improved in the clinical pharmacy learning path to ensure that it keeps pace with the evolution of pharmacists' clinical responsibilities in practice. Success factors for change in clinical pharmacy practice were also explored. Interviews were transcribed verbatim and analyzed thematically.

**Results:**

Success factors for advancing clinical pharmacy practice included elements related to the healthcare and political landscape, such as remuneration, leadership, as well as willingness and readiness for change. Other success factors identified are directly related to the pharmacist, such as the use of reflective practice or the pharmacist's motivation.

Perceived future directions include the existence of a multidisciplinary approach in practice, the expansion of clinical pharmacy services, as well as the delivery of clinical pharmacy services at the primary care level.

**Conclusion:**

The development of clinical pharmacy practice relies on a wide range of factors. The analysis carried out in this study of the success factors as well as the potential future directions of clinical pharmacy can serve as catalysts for the development of this pharmacy field at the country, regional, and global levels.

## 1 Introduction

Clinical pharmacy emerged in the early 1960s as a form of pharmacy practice and has been established as an evolutionary step in the practice of pharmacy following a deeply focused scientific and product‐oriented eras [1]. The European Society of Clinical Pharmacy states that *“Clinical Pharmacy aims to optimize the utilization of medicines through practice and research in order to achieve person‐centered and public health goals”* [2].

Clinical responsibilities are embedded in the practice of pharmacists in many countries, although there are differences between countries in the depth and quality of its application [3, 4], and they are included in all stages of the drug‐use process [5]. By reviewing drug therapies, assessing medicine‐related outcomes, performing therapeutic drug monitoring, and providing medicine information among other tasks, clinical pharmacists increase patient safety and medicines' effectiveness while decreasing healthcare costs [1, 6, 7].

The International Pharmaceutical Federation (FIP) in its Global Vision for Education and Workforce, calls all education, practice, and research leaders to join efforts in each country and region for the development of the profession [8]. The development and evolution of clinical pharmacy practice in each country always depend on existing facilitators and barriers, which are variable and highly context‐related, relying on various factors, including monetary, regulatory, technical, attitudinal, and political [9, 10].

As the profession has shifted towards prioritizing patient‐centred care and health outcomes, enabling pharmacists to focus more on clinical pharmacy, it is crucial to investigate the future scope of their role and possible success factors that enable change in practice.

Numerous studies have explored the provision of clinical pharmacy services, their outcomes, and the essential factors for their implementation. However, there is a notable absence of literature that portrays the point of view of experts worldwide on the future of pharmacists' clinical duties and the factors that motivate change in practice and development of clinical pharmacy.

By conducting semi‐structured interviews, this study aimed to explore the perspectives of global experts around the development of clinical pharmacy practice, with a specific focus on exploring success factors and their vision on the future of clinical pharmacy.

## 2 Methods

### 2.1 Study Design

A qualitative research approach was chosen as it enables the collection and analyses of data that are not easily captured by other approaches. Using semi‐structured interviews, researchers were able to gather objective data about the facts related to the development of clinical pharmacy in each country, while also delving into the interviewees' experiences and perspectives on the phenomenon [11].

### 2.2 Ethics

Ethical approval was obtained from the Ethics Committee for Research in Human Beings, Faculty of Pharmacy, University of Lisbon (Report n°04/2022).

### 2.3 Population Sample and Recruitment

The research team conducted a targeted literature review to address the question: “What is the current state of clinical pharmacy education and practice in the world?”. Before conducting the literature review, criteria were established in the domains of education, practice, and specialization to identify extraordinary advancements in clinical pharmacy. The criteria for education were i. existence of specific postgraduate education in Clinical Pharmacy (*e.g.,* MSc in clinical pharmacy), ii. existence of undergraduate education with a proven high‐level clinical component (*e.g.,* mandatory clinical rotations throughout the curriculum). For practice, the criteria included: i. development of pharmaceutical care plans in complex patients by pharmacists, ii. existence of independent prescribing by pharmacists, and iii. development of advanced medication review (type 3) by pharmacists. Additionally, any country that had implemented a specialization in clinical pharmacy was considered to have made significant progress in the field. Accordingly, the countries that, through the literature review, proved to fulfil at least three criteria, one of which had to be in the field of education and another in the field of practice, were selected to be analyzed. Australia, Canada, China, Czechia, Malta, Slovenia, the United Kingdom, and the United States, were then selected to be interviewed.

Experts from academia and different areas of clinical practice, who possessed strong knowledge of both the national context and the international landscape, were contacted via email and invited to participate in the study as interviewees. The expert sample was purposively selected based on the research team's network of international contacts. All the interviewees were internationally renowned experts in the field of clinical pharmacy, with scientific publications on the subject. If the initial contact did not consider him/herself the most appropriate expert, he/she recommended someone more suitable for the interview, according to the pre‐established criteria.

Following the initial interviews, a snowballing method was employed to uncover additional countries where clinical pharmacy practice and education have extraordinary advancements but were not previously identified in the literature review. This process led to the inclusion of five additional interviewees representing: Belgium, Japan, Malaysia, Saudi Arabia, and the Netherlands.

In the case of the Netherlands and Malaysia, the person initially contacted referred the research team to someone else considered more suitable to interview as part of the study. In the case of Slovenia, the person initially contacted was unavailable and thus had to be replaced by another expert.

The sample size was not fixed in advance but was guided by the sampling strategy and the assessment, based on data analysis, of the point at which saturation of themes was reached. Furthermore, two other factors were considered: the richness of the data and practical concerns, such as time limitations and the volume of data.

### 2.4 Data Collection

Semi‐structured interviews were conducted through Microsoft Teams version 24033.813.2773.520. Each interview was recorded in both audio and video format, with prior permission secured from the participants and formalized through a written consent form.

One researcher (LM) developed the interview guide, which was then verified by another researcher (FAC), adhering to best practices for qualitative research [11].

The guide was composed of open‐ended questions and included: “Do you think something has to be improved in the clinical learning path to provide clinical pharmacy services and/or to keep pace with new evolutions/roles of pharmacists in this regard?” and “What do you think might be the future of clinical pharmacy education and practice?”. Throughout the interview, further exploration of the first question was conducted through probes and prompts, delving into specific factors that facilitate change in clinical pharmacy practice.

Respondents were given prior access to the questions via e‐mail.

Data on countries' sociodemographic and workforce characteristics were obtained resorting to international databases, including information on population size and the ratio of pharmacists per 10,000 population. Additional data, such as, the predominant healthcare financing method and the healthcare innovation index ranking, were sourced from relevant literature.

### 2.5 Data Analysis

Interviews were transcribed *verbatim* and analyzed thematically. The transcriptions were conducted by one researcher (AC) and reviewed by another researcher (LM). One researcher (LM) was responsible for conducting the analysis, and findings were shared with the research team for verification (SNL and FAC).

Inductive and deductive approaches were used to conduct content in an iterative process until consensus was reached [12]. Thematic analysis was carried out by: identifying all relevant concepts and themes; indexing highlighted excerpts by theme and correlating them with others; rearranging the highlighted excerpts while considering their contexts to create a coherent and understandable flow; mapping the concepts, scope, and nature of the studied phenomenon; and attempting to associate themes to build an explanation for the findings, connecting them to preview literature [11, 13].

Both transcription and analysis processes were conducted manually, without the use of any software.

### 2.6 Positionality Statement

The researchers' background includes clinical practice, academia, and professional bodies institutions, from three countries (two different world regions), and engagement within the pharmaceutical workforce development policies and studies. These backgrounds were relevant to the definition of stakeholders around the world and the inductive approach during the analysis process, as the experiences in global debates in clinical pharmacy services and policies development informed rationale to the thematic identification. Nevertheless, methodological preparedness and ethics commitment with the interviewees guarantee the fairness of the results.

Only one researcher (LM), who is a PhD candidate, had direct contact with the interviewees, avoiding personal influence of the researchers during the interviews.

At the interviewees' request, the transcript was forwarded to them for verification of its content and its fidelity.

## 3 Results

The group of experts included nine females and four males. Ten respondents were affiliated with academic settings, while three had practical experience in addition to academic backgrounds.

The country profiles are available in Table 1.

**Table 1:** Country profiles.

Country	Direct access to perform clinical activities after pharmacy graduation	Population (in thousands)^a^	Pharmacists per 10 000 population^b^	Healthcare expenditure per capita (US $)^a^	Predominant method of financing healthcare c, d	Healthcare innovation index (2022)^e^, ^f^
Australia	Yes	25,921 (2021)	10.44 (2020)	7055 (2021)	National health insurance	53.29 (Good)
Belgium	Yes	11,555 (2021)	20.27 (2021)	5680 (2021)	Bismarck	51.99 (Good)
Canada	Yes	38,454 (2022)	10.55 (2021)	6207 (2022)	National health insurance	43.69 (Moderate)
China	Yes	1,425,893 (2021)	3.21 (2017)	671 (2021)	Out of pocket	No data available
Czechia	No. Specialization in clinical pharmacy is needed.	10,702 (2021)	7.21 (2020)	2499 (2021)	Bismarck	45.97 (Moderate)
Japan	Yes	124,613 (2021)	20.00 (2020)	4347 (2021)	Bismarck	43.64 (Moderate)
Malaysia	Yes	33,574 (2021)	3.38 (2015)	487 (2021)	Bismarck	No data available
Malta	Yes	516 (2021)	25.48 (2021)	3642 (2021)	Beveridge	No data available
Netherlands	Yes	17,475 (2021)	2.17 (2020)	6539 (2021)	Bismarck	59.86 (Elite)
Saudi Arabia	No. Postgraduate education in clinical pharmacy is needed.	35,950 (2021)	8.58 (2021)	1442 (2021)	Beveridge	37.30 (Poor)
Slovenia	No. Specialization in clinical pharmacy is needed.	2107 (2022)	7.35 (2020)	2608 (2022)	Bismarck	No data available
United Kingdom	Yes	67,791 (2022)	8.53 (2020)	5139 (2022)	Beveridge	49.24 (Good)
United States of America	Yes	338,290 (2022)	10.64 (2020)	12,474 (2022)	Out of pocket	50.66 (Good)

^a^ Global Health Expenditure Database—World Health Organization.

^b^ The Global Health Observatory—World Health Organization.

^c^ Alfaro M, Muñoz‐Godoy D, Vargas M, et al. National Health Systems and COVID‐19 Death Toll Doubling Time. Front Public Health. 2021;9:669038. Published 2021 Jul 15. doi:10.3389/fpubh.2021.669038.

^d^ Thompson S, Foubister T, Mossialos E. Financing health care in the European Union, challenges and policy responses. In: Observatory Studies Series 17. European Observatory on Health Systems and Policies. Copenhagen: WHO Regional Office for Europe; 2009.

^e^ Cravo Oliveira T, Barrenho E, Vernet Erkko Autio A, Barlow J. Developing a Global Healthcare Innovation Index; 2017.

^f^ Roy, A. Key Findings from the 2022 World Index of Healthcare Innovation. Medium website. https://freopp.org/key-findings-from-the-2022-world-index-of-healthcare-innovation-e2a772f55b92. Published March 16, 2023. Accessed February 22, 2024.

The analyzed countries exhibit significant variations in population size, the quantity of practicing pharmacists, and the level of investment in health. The countries represented a diverse range of health financing systems, with the Bismarck model that relies on an insurance framework typically funded through contributions from both employers and employees via deductions from their payrolls [14] being the most widely adopted among them. The Beveridge model, where healthcare is funded by the government through taxation [14], is the following most common financing model of the analyzed countries. Additionally, countries have moderate to elite scores on the healthcare innovation index, except for Saudi Arabia, which scores poorly on the subject [15].

Access to clinical practice is direct upon graduation from the undergraduate pharmacy program in most countries, except for Czechia, Saudi Arabia, and Slovenia, where pharmacists need to undergo postgraduate training before being allowed to perform clinical pharmacy activities autonomously.

**Figure 1:**
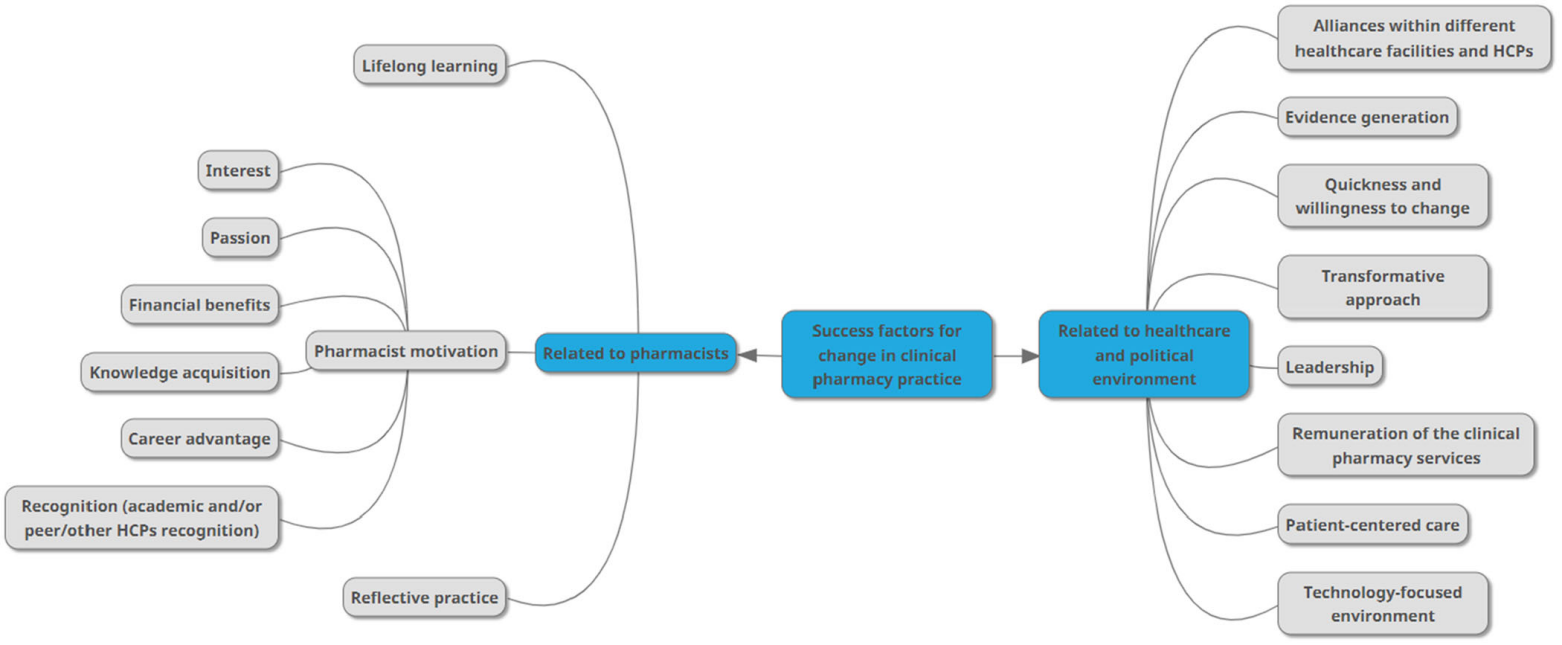
Visual representation of the codes related to success factors for change in practice in the views of the interviewees.

**Figure 2:**
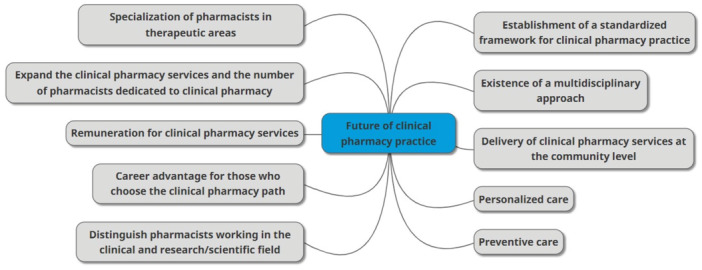
Visual representation of the codes related to the future of clinical pharmacy practice in the views of the interviewees.

From the analysis of the interviews, two main thematic categories arose exploring the views of the interviewees around: i. the success factors for change in clinical practice (Figure 1) and ii. the future of clinical pharmacy practice (Figure 2).

### 3.1 Success Factors for Change in Practice

Respondents identified several success factors that promote change in clinical pharmacy practice, based on their experiences and perceptions.

A full list of the factors along with all representative quotes is outlined in Table 2.

**Table 2:** Success factors for change in practice.

Themes		Representative quotes
Related to the healthcare and political environment		
Leadership		*“It's been very slow, so I would have made that happen faster…//…It's about leadership within the NHS system”*
		(UK respondent, female, Academia)
Willingness and readiness for change		*“…//… and the willingness to change and the money to change [within the NHS]”*
		(UK respondent, female, Academia)
		*“Now because the healthcare system is so stretched, and we need healthcare providers of any training who can practice to their full scope. I think society is asking pharmacists to do more and there's been a pull. As much as there's a push, there's a pull. So, now society is pulling pharmacists to come and help do many things that we need.”*
		(Canada respondent, female, Academia)
Remuneration of the clinical pharmacy services		*“It's up to your decision if you want to be on full‐time equivalent or only part‐time equivalent clinical pharmacist but it's a paid job”*
		(Czechia respondent, female, Academia and Practice)
		*“We provide seamless care…//…which is extra paid to reimburse all Slovenian hospitals. So we get €14.00 per case to make medication reconciliation at admission to make the best possible medication history first and then medication reconciliation admission to make the reconciliation with a doctor.” and “We have to provide a medication review and then this medication review goes to GPs and then they decide. And this service is paid by National Slovenian Insurance 50€ per case. And if they have 10 or more medications, we are paid 100€ per patient, this is a gross income”*
		(Slovenia respondent, male, Academia and Practice)
Patient‐centered care		*“The main point is to be focused on the patient. To be focused on patient outcomes and clinical experiences. Without that, you cannot expect success.”*
		(Slovenia respondent, male, Academia and Practice)
Technology‐focused environment		*“Consideration of technology and sort of how to work in an ever‐increasingly technology‐focused environment.”*
		(Canada respondent, female, Academia)
Transformative approach		*“We are trying to prepare people to be transformative and you can be transformative by learning how others are doing …//… networking on a European and global level is another thing that needs to be built”*
		(Malta respondent, female, Academia)
Evidence generation		*“[in our studies] We show that this is a very important service and the number of medications in total reduces by 10%, the number of DDIs by 50%, and the number of PIMs by 30%. This collaboration led to a better quality of life which is an important humanistic outcome, not just the number of medications. And the return on investment was 3 to 1 and 5 to 1, which means that this is a very good cost‐effectiveness approach too…//… I'm heavily involved in Slovenia…//…negotiations…//… with politicians…//… [to show them] how and why we are important. This is very important for the future of clinical pharmacy and without that, we don't have advanced clinical pharmacy services.”*
		(Slovenia respondent, male, Academia and Practice)
		*“If all goes well and we demonstrate a positive impact and how they can contribute to the patients meeting those metrics, those quality metrics which in turn they mean that the health system gets more money because they are reimbursed on a pay‐for‐performance mechanism, well, they get paid or they get the reward.”*
		(USA respondent, female, Academia)
Alliances within different healthcare facilities and HCPs		*“You must be a good clinician and you must be a good partner of physicians because they are usually asking for resolving problems that they cannot resolve…//… you must be able to establish interdisciplinary cooperation and, in the system, when you are not paid you must be good to convince your collaborating physicians that you are important for them. And to convince them that they should want you and they probably should pay you or help you to get payments for your job.”*
		(Czechia respondent, female, Academia and Practice)
		*“China is a huge country, and the pharmacy services are mainly in larger hospitals and urban areas…//… the hospitals are beginning to form alliances, so a larger hospital works with a smaller hospital. Because in a larger hospital, you have strong clinical pharmacy services, so those services can provide help to the low‐tier hospitals.”*
		(China respondent, male, Academia)
Related to pharmacists		
Lifelong learning and Reflective practice		*“It's making everybody into the sort of learner who is always learning, and I think things like revalidation of practice make people do that…//…but the moment we have to do reflections, we have to do peer review, we have to document some learning every year.”*
		(USA respondent, female, Academia)
		*“The health professions are like lifelong learning”*
		(USA respondent, female, Academia)
Pharmacist motivation	Interest and Passion	*“In terms of “why they do it”, they typically do it just may be of interest. There are no benefits in terms of salary or remuneration, so it is more because of the passion and their interest and also for work‐related advancements because they want to improve their work.“*
		(Malaysia respondent, male, Academia)
		*“Clinical pharmacy is the one for those who are very brave and really want to be clinicians”*
		(Czechia respondent, female, Academia and Practice)
	Financial benefits	*“In a hospital pharmacy, you definitely get that money, you will earn more”*
		(Netherlands respondent, female, Academia)
		*“Specialists from clinical pharmacies are approximately 40% more paid than the pharmacist in the hospital”*
		(Slovenia respondent, male, Academia and Practice)
		*“And then in Wales and Scotland, they've trained a lot more clinical independent prescriber pharmacists and paid for them to be prescribers within the pharmacy contract in Community Pharmacy and England's behind on that…//… so they have additional rights”*
		(UK respondent, female, Academia)
	Knowledge acquisition	*“In a hospital pharmacy, you definitely…//…are higher skilled”*
		(Netherlands respondent, female, Academia)
		*“You get a lot of respect and recognition from peers as well from the doctors when we do clinical rounds because of the knowledge and also because of the expertise”*
		(Malaysia respondent, male, Academia)
	Career advantage	*“The career development there, the career path, is really good”*
		(Netherlands respondent, female, Academia)
	Recognition (academic and/or peer/other HCPs recognition)	*“You get a lot of respect and recognition from peers as well from the doctors when we do clinical rounds because of the knowledge and also because of the expertise, the wealth, and also the sharing that pharmacists can bring in. So, they value this pharmacist as part of the healthcare team.”*
		(Malaysia respondent, male, Academia)

The healthcare and political environment, such as leadership, willingness and readiness of the system and health care professionals to change, and the decision of providing remuneration for clinical pharmacy services constitute an important part of the success factors for clinical pharmacy implementation. The increasing technological development and the health care model were also stressed as success factors, considering the patient‐centred care model an opportunity to the development of clinical pharmacy services. Also, the evidence produced by the clinical pharmacy services is recognized as crucial foundation to boost and sustain those services within the health system.

In addition, other recognized factors are directly related to the pharmacist and the pharmacy profession. Professional motivation is reported as a key factor for the success of clinical practice. It is expressed through concrete conditions, such as better remuneration and opportunities to take advantage of the clinical pharmacy career. The interest and passion for pursuing a clinical pharmacy career path were observed by interviewees as part of the scenario of success for practice change.

### 3.2 Future of Clinical Pharmacy Practice

Ten themes emerged from the analysis of the respondents' perspectives on the future of clinical pharmacy practice. Table 3 displays all the codes and the representative quotes.

**Table 3:** Future of clinical pharmacy practice.

Themes	Representative quotes
Expand the clinical pharmacy services and the number of pharmacists dedicated to clinical pharmacy	*“One of our aims is to have even more of these clinical pharmacists, not only in acute wards but also in GP ambulances, nursing homes, in‐home care, hospitals, in different settings of care.”*
	(Czechia respondent, female, Academia and Practice)
	*“I really hope and think that this caregiver role, this clinical role for pharmacists needs to be more fundamental and needs to be available throughout the country”*
	(Netherlands respondent, female, Academia)
	*“We are discussing with the GPs about independent prescribing here in these primary care centers”*
	(Slovenia respondent, male, Academia and Practice)
Existence of a multidisciplinary approach	*“[the future should include a] Multidisciplinary approach and combining theory with practice”*
	(Belgium respondent, female, Academia and Practice)
	*“[the future will be] Much more focused on therapeutic decision‐making and providing services and care as part of …//… groups of interprofessional teams…//… and the pharmacist would be one of the networks of providers that would be involved in helping people with their health.”*
	(Canada respondent, female, Academia)
	*“I would like to see more collaborative practice between pharmacists and physicians”*
	(China respondent, male, Academia)
	*“[there is a continuous need] To have this collaboration with other healthcare professionals.”*
	(Czechia respondent, female, Academia and Practice)
Personalized care	*“Patient centricity and patient needs, you know, in that patient centricity, the patient needs going all along. We talk a lot about personalization, personalized medicine, and personalized care.”*
	(Malta respondent, female, Academia)
Establishment of a standardized framework for clinical pharmacy practice	*“I think the system has to define the role of a clinical pharmacist in practice and have an established framework for practicing in the clinical pharmacy sector.”*
	(Saudi Arabia respondent, female, Academia)
Remuneration for clinical pharmacy services	“*I think further demonstrates the impact of pharmacy and we're working with a large health system. So, if all goes well and we demonstrate a positive impact and how they can contribute to the patients meeting those metrics, those quality metrics which in turn they mean that the health system gets more money because they are reimbursed on a pay‐for‐performance mechanism, well, they get paid or they get the reward.”*
	(USA respondent, female, Academia)
Career advantage for those who choose the clinical pharmacy path	*“I think in the future there will be a career advantage because you'll get better jobs eventually”*
	(Australia respondent, female, Academia)
Preventive care	*“I think that [the future] will evolve to have more of a preventative health component”*
	(Canada respondent, female, Academia)
Delivery of clinical pharmacy services at the primary care level	*“One is going to take up a position in a community pharmacy one can provide not only the dispensing part but also the clinical services.”*
	(Malta respondent, female, Academia)
	*“I think that [the future] will evolve to have more…//… of a community care‐based component”*
	(Canada respondent, female, Academia)
	*“The clinical pharmacy services are heavily centered in larger hospitals and in larger cities. What they should do is to provide those services at a community level that would have a bigger impact on population health. I think that they should develop that future clinical pharmacy practice at a community level.“*
	(China respondent, male, Academia)
Specialization of pharmacists in therapeutic areas	*“I think there is a big potential in terms of …//… specialization because currently, we are producing a lot of generalists, at least in this part of the world.”*
	(Malaysia respondent, male, Academia)
Distinguish pharmacists working in the clinical and research/scientific field	*“I think that this is also the future, that it will be a separate clinical field and scientific field.”*
	(Czechia respondent, female, Academia and Practice)

The existence of a multidisciplinary approach in practice, the expansion of clinical pharmacy services, and the number of pharmacists dedicated to clinical pharmacy, as well as the delivery of clinical pharmacy services at the primary care level were the themes identified by a greater number of countries.

### 3.3 Clinical Pharmacy Services

Additionally, throughout the interviews, the respondents highlighted some clinical pharmacy services available in their countries (Table 4).

**Table 4:** Clinical Pharmacy services available.

Themes	Representative quotes
Clinical rounds	*“We make fast rounding from bed to bed”*
	(Slovenia respondent, male, Academia and Practice)
Reporting of clinical observations to other HCPs	*“When I have a patient I ask them, and they give me feedback, and usually, they give me much more information than when we make fast rounding from bed to bed…//… I can report all my observations and my recommendations to the doctors at our meeting the next day”*
	(Slovenia respondent, male, Academia and Practice)
Independent prescribing	*“In Wales and Scotland, they've trained a lot more clinical independent prescriber pharmacists and paid for them to be prescribers within the pharmacy contract in Community Pharmacy”*
	(UK respondent, female, Academia)
Prescription renewal	*“Originally we started with every two months, the patient had to go to the prescriber because pharmacist prescribing was not allowed. So, by the end, this was developed so that the pharmacist can review the medication and renew that prescription.”*
	(Malta respondent, female, Academia)
Medication review	*“We have to provide a medication review and then this medication review goes to GPs and then they decide*. *And this service is paid by National Slovenian Insurance 50€ per case. And if they have 10 or more medications, we are paid 100€ per patient, this is a gross income”*
	(Slovenia respondent, male, Academia and Practice)
	*“Home medicines review, which is one of our, I think, flagship clinical services”*
	(Australia respondent, female, Academia)
	*“[the pharmacists] Are responsible for ensuring people are educated and doing appropriate therapeutic workups of patients including, interviewing to understand the patient's condition and context, assessing the medication choice and medication prescriptions and educating the patient and working as part of interdisciplinary teams to help people with their health”*
	(Canada respondent, female, Academia)
	*“We have patient medication reviews in community practice for chronic medications”*
	(Malta respondent, female, Academia)
Medication reconciliation	*“Concerning medication reconciliation, we are in the emergency department every day to have mitigation lists accurately registered”*
	(Belgium respondent, female, Academia and Practice)
	*“We get €14.00 per case to make medication reconciliation at admission to make the best possible medication history first and then medication reconciliation admission to make the reconciliation with a doctor”*
	(Slovenia respondent, male, Academia and Practice)
Consultation services	*“That is why they recently created the home pharmacy system, where community pharmacists have a consultation within 24 h in the community pharmacy.”*
	(Japan respondent, male, Academia)
Therapeutic drug monitoring	*“And we also have TDM, therapeutic drug monitoring”*
	(Belgium respondent, female, Academia and Practice)
Referral to other levels of care/HCPs and Chronic diseases monitoring	“W*e started to have projects, we had research projects where we were seeing if the pharmacist had the services and follows blood pressure, blood glucose, and lipid profile every three months for our diabetic patients. What were the outcomes? And when you showed the outcomes that these patients were in better range because they were referred, then the community starts to become more open.”*
	(Malta respondent, female, Academia)
Patient education	*“We teach the patients at the discharge date for example, how they should take medication, and why they have these medications, and we can also dispense medication for one month.”*
	(Slovenia respondent, male, Academia and Practice)
	*“We also have many ambulatory patients who require specific medication that is only delivered by the hospital pharmacy, so, not in the outpatient pharmacy. So, we also tried to counsel these patients on how to take these medications, and what about their medication list”*
	(Belgium respondent, female, Academia and Practice)
	*“[the pharmacists] Are responsible for ensuring people are educated and doing appropriate therapeutic workups of patients including, interviewing to understand the patient's condition and context, assessing the medication choice and medication prescriptions and educating the patient and working as part of interdisciplinary teams to help people with their health”*
	(Canada respondent, female, Academia)
Management of patients through social media	*“China is a huge country, and the pharmacy services are mainly in larger hospitals and urban areas. What they started to do is manage patients on social media. For instance, if you manage anticoagulation clinics they establish a group chat so the patients can ask questions. They also developed apps where you can reach the pharmacist and the pharmacist can reach to the patient”*
	(China respondent, male, Academia)
Identify and act upon drug interactions and adverse drug reactions	*“We have to compile the list again and perform some interaction screening. Also, when the patient returns, how does it go? Adherence and all things like that, as patients suffer from adverse drug reactions, so, the counseling of ambulatory patients with regard to specific medication is also a back‐office clinical pharmacy task”*
	(Belgium respondent, female, Academia and Practice)
Management of complex patients	*“You must be trained in these interventions in highly complex patients or high‐risk patients and then you must be able to conduct prospective research or even retrospective if using some hospital data, lab tests, and all these clinical information.”*
	(Czechia respondent, female, Academia and Practice)

The most reported clinical pharmacy service was medication review, followed by patient education. The management of complex and high‐risk patients was also reported as a differentiating and upskilled clinical pharmacy service.

## 4 Discussion

The progression of clinical pharmacy practice in the sampled countries has been shaped by several success factors, including socioeconomic conditions, the health care system, the workforce, and pharmacy education available. These factors have led to a wide range of scenarios, experiences, and expectations reported by the interviewees.

The reported factors related to the success of the implementation of clinical services are aligned with results of previous studies focused in different contexts [16]. An important challenge consistently at the forefront of debates around clinical services was remuneration. Remuneration involves structuring the health system, whether public or private, to acknowledge the value and the relevance of these services. Addressing this challenge requires research, systematic monitoring the results of service outcomes, information technology, and strong advocacy efforts.

The fact that countries have identified themes such as lifelong learning, financial investment, and willingness and readiness for change is particularly interesting since the education of the human capital, the monetary investment, and the political and regulatory environment are weighting factors when calculating each country's score for the healthcare innovation index. Indeed, the fact that the respondents identified these as success factors may be related to the good scores of these countries on this index [17].

Pharmacists' openness and motivation to perform clinical pharmacy services and embrace practice changes were identified as critical facilitators. Over the last two decades, pharmacists have demonstrated desire to shift their practice towards activities with greater clinical focus. However, the fulfilment of these efforts depends on working conditions, opportunities, regulation, and recognition. Although motivation is a critical success factor for the development of clinical pharmacy practice, it is not merely an individual trait, but rather a collective, contextual condition [18].

Countries such as Czechia and Slovenia, where pharmacists need to complete postgraduate training to carry out clinical pharmacy activities, emphasize remuneration for clinical pharmacy services as a success factor for practice development. This may suggest that, since clinical services are seen as a differentiation from a pharmacist's normal activity in these countries, such services are considered added value and include a specific remuneration or are directly included in the pharmacist's salary. Perhaps the fact that these countries have Bismarck as the predominant financing model has had an influence since countries that have implemented this model tend to have a greater financial investment in health [14], and perhaps a greater culture for evidence‐based decision making. The generation of evidence around the outcomes of clinical pharmacy activities as a means of negotiation to achieve funding for certain clinical pharmacy services was also identified by respondents from Slovenia and the USA. These results are consistent with the existing literature on payment for clinical pharmacy services in these countries [4, 19]. A study that provides recommendations for the wider adoption of clinical pharmacy in Central and Eastern Europe highlights the need for enabling and supporting clinical pharmacy research as a contributing factor [4].

From the interviewees' perspective, the future of clinical pharmacy should be geared towards community‐based clinical pharmacy services provision and the enhancement of multidisciplinary collaboration in the healthcare provision. Both themes were mentioned by respondents from several countries, regardless of their health financing model. This vision fulfils the aim of the Astana Declaration to strengthen primary healthcare in a multidisciplinary context, allowing equitable and close access to healthcare for all [20]. The vision for greater and more effective multidisciplinary collaboration will certainly be easier to achieve when the basis of education is already focused on interdisciplinary education, which is still not adequately consolidated in the field of pharmacy [21].

Expansion of clinical pharmacy services and the number of pharmacists working in clinical pharmacy were identified as priorities by Czechia, Slovenia, the Netherlands, and the USA. All these countries, except for the USA, have a low rate of practicing pharmacists per population when compared to the remaining analyzed countries. Additionally, for Czechia and Slovenia, which consider clinical pharmacy as a specialization area within the profession, this vision is much more relevant. These topics all seem to be connected since a greater number of pharmacists allow increased clinical pharmacy implementation by devoting more time to the development of clinical services within the pharmacists' responsibilities. Increasing numbers of well‐prepared clinical pharmacists providing added‐value clinical activities enable recognition of the value of these services by the population and other healthcare professionals (HCPs). In turn, recognizing the value of the clinical services provided by pharmacists will bring more investment, which allows the expansion of clinical activities carried out by pharmacists at all levels of care, turning it into a development cycle.

Effectively translating research into everyday clinical practice and the formulation of policies, known as implementation science, is also crucial for advancing clinical pharmacy practice [22]. A systematic review of existing clinical pharmacy guidelines around the world highlighted that most existing guidelines emerged after 2016. Thus, the expansion and regulation of clinical pharmacy services has been an established reality, although limited to a set of English‐speaking countries such as Australia, Ireland, the UK, and the USA [23]. Moreover, the ambition of having an increase in the number of pharmacists is aligned with the published literature [9].

Respondents mentioned some of the clinical pharmacy services implemented in their country, the most frequently mentioned being medication reviews. Published literature indicates that medication review is a crucial responsibility for pharmacists, yielding significant benefits in optimizing therapy, particularly advanced or type 3 medication review [24, 25]. The implementation of medication review is on the rise at the different levels of healthcare, which aligns with the fact that it was also the clinical pharmacy service most frequently mentioned by the interviewees.

The development and investment in a certain type of service shall be closely related to the specific needs of the country and the population. For example, independent prescribing by pharmacists and other nonmedical professionals emerged in the UK due, but not limited, to a lack of available physicians in the country to meet the prescribing needs [26]. Another example is the management of patients through digital tools such as social networks by pharmacists in China, which might be related to the huge Chinese territory, the low number of practicing pharmacists per 10,000 inhabitants, and the difficulty of guaranteeing healthcare close to the entire population [27].

Findings emphasize that countries evolve differently. A clear example of this is the existence of specializations in therapeutic areas. Interestingly, countries such as the USA and the UK have been committed to the possibility of pharmacists acquiring specific expertise in therapeutic areas for some time [28, 29], while other countries, such as Malaysia, mention it as a necessity for the future, and where clinical pharmacists are now more generalists. Another example is the benefits of career development, which for the Netherlands is a success factor for motivating pharmacists, and for Australia is still an ambition for the future. In general, pharmacists' career development, including education, lifelong learning, and recognition, is essential for enhancing the availability, quality, and impactful growth of clinical pharmacy services [9].

A limitation of this study arises from the diverse contexts and variations in clinical pharmacy practices among countries worldwide, making comparisons challenging. Nonetheless, to enhance the analysis of the collected data, contextual research was conducted as presented in Table 1. Also related to this variability in contexts, the term clinical pharmacy and what clinical pharmacy services are can be interpreted in different ways by countries and respondents, a limitation that we tried to mitigate by starting with a clear definition of the concept adopted, followed by the selection of interviewees with extensive knowledge in the field and international experience.

Other limitations of the study pertain to the sample of countries included. The research team recognizes that the literature review conducted might have restricted the selection of countries for interviews, potentially excluding some countries that also have extraordinary practices in clinical pharmacy but may publish less or publish in PubMed non‐indexed journals (publication bias). The research team tried to mitigate this limitation by using a snowballing approach to find other countries with good practices that had not passed the screening of the literature review. Conversely, the use of this snowballing approach is always prone to an increased degree of subjectivity in the choice of countries. Additionally, the sample was created by convenience, which may have biased the results since these are individuals who are more involved with the international community and therefore have a more global perspective but may not necessarily be the national champions. Another limitation is that not all world experts could be interviewed, even though we tried to get a wide sample.

Future studies could assess the impact of each of the themes identified from the perspective of a wider sample of pharmacists within each country or a wider sample of countries focusing on non‐English speaking countries that may be champions within their regions. Additionally, analyzing differences in the development of clinical pharmacy practice according to the health financing system of each country could be an interesting area of investigation.

Analyzing the state of the art and the future of clinical pharmacy practice through the eyes of experts from around the world is an important step in moving this area of pharmacy practice forward. These experts are drivers of evolution and influence within their own countries but also internationally. Learning from their experiences and expectations gives some important directions for consolidating clinical pharmacy services in diverse scenarios. The investment in pharmacy workforce and career development, qualification, and recognition are core factors. It is not feasible to plan to grow clinical services without expanding, educating, and training the pharmacy workforce.

Nevertheless, the reported experiences suggest that the health system model and the regulatory environment can have a strong influence on the conditions for the development of clinical pharmacy services. This suggests that the development and consolidation of clinical pharmacy services in the future relies on broad movements to develop the right to access medicines and health services, aside with the competencies to use medicines in a responsible and informed manner, supported by pharmacists. This involvement must include interventions in the political field (in formulating and implementing public policies), the social field (offering services, information and patient advocacy), the scientific field (investing in research that provides evidence on the validity and effectiveness of pharmacy pharmaceutical services) and the technical field (establishing services that actually meet the population's health needs).

Therefore, the findings of this study enhance the understanding of the success factors in implementing and developing clinical pharmacy globally and provide insights into the future of the clinical aspect of pharmacist practice. In this way, they can play a role in shaping a vision for clinical pharmacy practice on both national, regional, and global scales. Moreover, they can aid in developing the appropriate policies to ensure the sustained evolution of this field in the pharmacy landscape.

## Conflicts of Interest

The authors declare no conflicts of interest.

## Data Availability

The data that support the findings of this study are available from the corresponding author upon reasonable request.
